# Female behavioral strategies during consortship in Tibetan macaques (*Macaca thibetana*)

**DOI:** 10.1002/ece3.7790

**Published:** 2021-06-22

**Authors:** Qi‐Xin Zhang, Lixing Sun, Dong‐Po Xia, Jin‐Hua Li

**Affiliations:** ^1^ School of Resources and Environmental Engineering Anhui University Hefei China; ^2^ International Collaborative Research Center for Huangshan Biodiversity and Tibetan Macaque Behavioral Ecology Hefei China; ^3^ Department of Biological Sciences Central Washington University Ellensburg WA USA; ^4^ School of Life Sciences Anhui University Hefei China; ^5^ Schools of Life Sciences Hefei Normal University Hefei China

**Keywords:** concealed ovulation, consortship, female behavioral strategies, Tibetan macaques (*Macaca thibetana*)

## Abstract

Consortship has been defined as a temporary association between an adult male and an estrous/receptive female. It has been considered as male mating strategies to improve male mating success and potential reproductive success. However, the female roles have been more or less neglected, and thus, less is known about female behavioral strategies during the consortship periods. In this study, during the two consecutive mating seasons, we collected behavioral data of free‐ranging Tibetan macaques (*Macaca thibetana*) habituated in Mt. Huangshan, China, to investigate female behaviors when she was consorted by an adult male. The results showed that (a) females were more likely to approach and exhibit sexual solicitation to their consorting males during the consorted period, and females also exhibited less approach to their nonconsorting males; (b) females exhibited strong responses (either departed distantly or formed affiliative relationships with their consorting male partner) when their consorting males mated with rival females or showed sexual motivation toward rival females; (c) female preferences were positively correlated to the duration of consortships and the frequencies of ejaculation copulations, independent of the social ranks of their consorting male partners. Our results suggested that female strategies played much more important roles in forming and maintaining consortship than previously assumed. It provides new insight into understanding female adaptive strategies to male strategies by forming consortships in multimale–multifemale primate species when males could not identify female's fertile phase accurately.

## Graphical abstract

The study is relevant in today's society and should be of interest to a wide readership. The contribution of the study and the wider implications of the results are clearly presented and discussed in the manuscript.

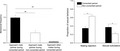

## 1 INTRODUCTION

Consortship is generally described as a temporary association between an adult male and an estrous/receptive female, characterized by the couple's close proximity and coordinated movements (Carpenter, 1942; Huffman, 1987, 1992; Manson, 1997; Rakhovskaya, 2013; van Noordwijk, 1985). Consortship has been reported in many primate species (*Papio anubis*, Packer, 1979; *Macaca mulatta*, Hill, 1987, Rakhovskaya, 2013; *Papio cynocephalus ursinus*, Seyfarth, 1977, Noë & Sluijter, 1990; *Macaca fuscata*, Perloe, 1992, Soltis, 1999, Takahata, 1982, Huffman, 1992; *Papio hamadryas*, Weingrill et al., 2003, Crockford et al., 2007; *Macaca sylvanus*, Brauch et al., 2008, Kümmerli & Martin, 2005; *Macaca assamensis*, Ostner et al., 2011; *Macaca tonkeana*, Rebout et al., 2017; and *Macaca fascicularis*, van Noordwijk, 1985; *Macaca radiata*, Shively et al., 1982), and thus, consortship was widespread in nonhuman primate society with multimales and multifemales. It is a long‐term adaptive consequence with selective pressure of ecological and evolutionary to facilitate the social relationship between a male and a female, to potentially affect male reproductive success in the promiscuous mating system (Engelhardt et al., 2006; Manson, 1997; Setchell et al., 2005; Tutin, 1979).

Previous studies suggested that consortship could be considered a male behavioral strategy to facilitate male mating efforts or male mate guarding (Summarized from Manson, 2017). In seasonal breeding primate species, the majorities of consortships were found to be significantly higher in the mating season (higher copulatory behavior with ejaculation) than those in the nonmating season (lower copulatory behavior with nonejaculation), such as Rhesus macaques (*Macaca mulatta*, Small, 1990), long‐tailed macaques (*Macaca fascicularis*, van Noordwijk, 1985), and Tibetan macaques (*Macaca thibetana*, Li & Kappeler, 2020). During consortship, an adult male was found to follow and frequently monitor (i.e., look in the direction of an individual) an estrous/receptive female, to synchronize with the daily activities of this female (Manson, 1997, 2017; van Noordwijk, 1985). Adult males benefited by forming consortship is to increase the mating opportunities with females consorted and that with other females nonconsorted (Manson, 1997). Several studies with paternity analysis demonstrated that consortship is a good indicator of male paternity. For example, in one research of *Mandrillus sphinx*, males sired 69% offspring during the consortship whereas males sired 31% offspring during the nonconsortship (Setchell et al., 2005).

In addition, consortship has also been considered as temporary intersexual social bonds, which contains two individuals, one adult male and one estrous/receptive female. For males, forming consortship could be an effective way to prevent other adult males from estrous females; however, females were not passive toward the consort behavior of adult males. Growing evidence suggested that adult females might also initiate adult males to form consortship. For example, in a study of long‐tailed macaques (*Macaca fascicularis*), females were observed to wait for the following consorted males or invite consorted male partners during the collective movement (van Noordwijk, 1985). Female acceptance may also facilitate forming consortship within intersexual dyads. For example, in a study of Assamese macaques (*Macaca assamensis*), when an adult male tried to consort an adult female (A), this male would move to another female (B) if the adult female (A) refused or flee (Sukmak et al., 2014). In Tonkean macaques (*Macaca tonkeana)*, there was evidence that female preference could influence whether an adult male could possessively consort an adult female by approaching and showing presentations to other males (Rebout et al., 2017). These case studies indicated that, apart from male behavioral strategies, female strategies also played crucial roles in forming and maintaining consortship. But still, little is known about how females excise their behavioral strategies and their effect on modifying male consort behavior.

In most *sinica*‐*arctoides* and *fascicularis* lineage species of genus *Macaca*, females were generally reported to conceal their ovulation (Thierry, 2007). Accordingly, it provides an ideal opportunity to investigate the effects of female strategies on forming consortship, because it is difficult for adult males to identify female's fertile status to form consortship (Dubuc et al., 2012; Li, 1999). In several primate species in multimale and multifemale groups, dominant males have preferential access to fertile females and sire a disproportionate number of offspring (reviewed in Di Fiore, 2003). For example, in Japanese macaques, the highest‐ranking male engaged in most copulations of the group but has no obvious advantage on reproductive success (Huffman, 1992). Similarly, in rhesus macaques, there is a strong correlation between male dominance rank and male mating success, but the highest‐ranking male did not sire the most offspring (Small, 1990). And thus, it was suggested that, by concealing their ovulation, females might better excise mate choice on certain males or polyandrous matings, and benefit from increasing their fertilization opportunities, confusing paternity, and improving the survivorship of offspring (Ostner et al., 2011; Paul, 2002).

**FIGURE 1:**
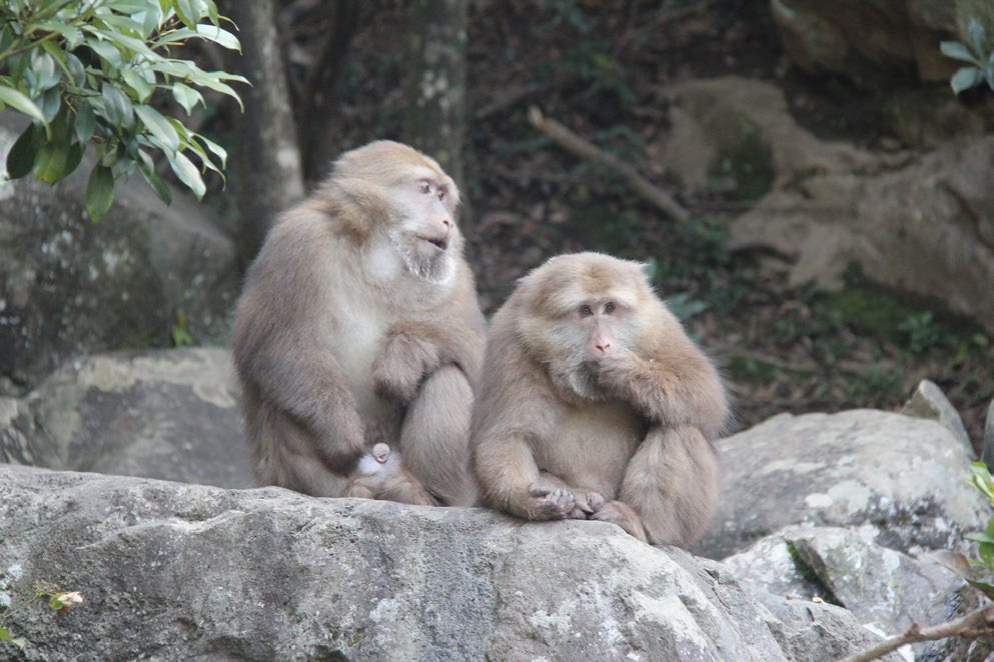
Consortship between the highest‐ranking male HXM (left) and a receptive female YXX (right)

In this study, we investigated the effects of female behavioral strategies on forming consortship in Tibetan macaques (*Macaca thibetana*). Tibetan macaques live in multimale and multifemale social groups (Li, 1999). Breeding is seasonal, with the mating season from July to January, and copulations occur during both the mating (higher frequency and with ejaculation) and nonmating season (lower frequency and without ejaculation) (Li et al., 2005, 2007). Females exhibit no obvious behavioral or visual signs of estrus (Li et al., 2005). Previous studies indicate that throughout the mating season, the alpha male engaged in 64% of copulations and the beta male 21% (see Xia et al., 2013). It remains unclear how reproductive successful dominant males are, relative to low ranking males in siring offspring, with the absence of data on paternity. High‐ranking males were found to frequently form consortship by following, co‐feeding, monitoring, and mating with consorted female partners during the mating season (Figure 1), whereas it was rare to observe that females initiated to form consortship (Li & Kappeler, 2020; Wu et al., 2018).

We compared the female behavior during consorted and nonconsorted periods, analyzed female responses to interactions between consort partners and other females, and compared the influences of female preference on consortship duration and mating frequency, to study female strategies during the consorted period. We tested the following predictions.

Assuming females prefer to form consortship with high‐ranking males to benefit from increased social tolerance and protection for their offspring, females would exhibit strong responses to male's consort behavior. Accordingly, we predicted that, within consortship dyads, consorted females would be likely to approach more frequently toward consorting male partners during the consorted period than during the nonconsorted period, or consorted females would approach more frequently toward consorting male partner than that toward other males. Consorted females would also participate in more sexual solicitations and less mating rejections toward consorting partners during the consorted period than during the nonconsorted period.

In addition, in seasonal breeding Tibetan macaques, female's receptive synchrony results in female intrasexual competitions. To enhance the competitive ability with rival females, females consorted by an adult male would also exhibit strong responses to rival females who were not being consorted by this male. And thus, we predicted that, a consorted female would maintain affiliative relationships with her consorting male, to decrease the opportunity of this male to mate with rival females. Moreover, if a consorting male had a chance to mate with or exhibit sexual motivation toward rival females, consorted female would depart distantly away from her consorting male to force this male following with her.

As such, if female efforts played crucial roles in maintaining consortship and benefited from these intersexual social bonds, we predicted that the duration of consortship would be longer, and the opportunities of mating would be higher during the periods that females formed consortship with their preferred males.

## 2 METHODS

### 2.1 Study site and subjects

We conducted this study in the Mt. Huangshan National Reserve, Anhui Province, China. The subject study group was Yulingkeng A1 (YA1). This group has been observed, and yearly demographic data have been continuously collected since 1986. This group inhabits a reserve known as the “Valley of the Wild Monkeys” (N30° 04′ 25.1″/E118° 08′ 59.3″) (Li & Kappeler, 2020). We can identify all adult individuals by natural markings and facial features.

All the behavioral data were collected across two consecutive mating seasons from August 2017 to March 2019. During the mating season from August 2017 to January 2018), the size of the study troop ranged from 46 to 48 members, including 15 adult females, 8–10 adult males, eight sub‐adults, six juveniles, and nine infants. On about 6 November 2017, the alpha male HXM was displaced by a young adult male, TRG. At the same period, two adult males, TG (originally second in rank) and YRQ (a young adult with the lowest rank) dispersed. During the mating season from July 2018 to January 2019, there were 49 members in this group including 15 adult females, nine adult males, six sub‐adults, seven juveniles, and 12 infants. On about 16 November 2018, the alpha male TRG was displaced by a natal young adult male, YCLO. Several nonresident adult males (one to four) were observed roaming peripherally toward the end of this period. As we could not easily identify them and they had little social interaction with the resident animals, we excluded them for analysis in the study.

### 2.2 Data collection

All the behavioral data were collected by a single observer (QXZ) from 08:00–12:00 and 13:30–17:30 while the focal subjects were in the forest (Altmann, 1974). We divided the daytime into four blocks (08:00–10:00, 10:00–12:00, 13:30–15:30, and 15:30–17:30) to distribute observation time evenly. We used ad libitum to identify consortship formed by an adult male and a female (Altmann, 1974). If a consort pair was identified during one block, the duration of consortship was recorded as one block (Li et al., 2005). Consortship was defined as an adult male and a receptive female observed sitting in proximity (within 5 m of each other) for at least 30 min and synchronizing their movements (i.e., following) twice or more, even in the absence of mating (Dubuc et al., 2012; Li et al., 2005; van Noordwijk, 1985).

A female was considered sexually receptive when she was observed mating with a male or with visible moist, pink genitals, sometimes with residual semen. Each receptive period was defined as a continuous period. Even if 1 or 2 days elapsed in which no mating activity was observed, they were included in the receptive period if mating resumed afterward (Dixon, 2012; Dubuc, Muniz, et al., 2012; Li et al., 2005). Female reproductive states were divided into three periods: (a) nonreceptive, when females did not show sexual receptivity; (b) nonconsorted, when females showed sexual receptivity but were not consorted by any males; and (c) consorted, when females showed receptivity and were consorted by a male.

The focal subjects were 15 adult females. We followed each randomly and continuously recorded their behavior for 30 min. Overall, we collected a mean of 10.07 ± 2.03 hr per female during the consorted period, 5.37 ± 0.88 hr during the receptive but nonconsorted period, and 17.48 ± 0.86 hr during the nonreceptive period. During each focal sampling, we also recorded the social interactions between the focal animal and other adult males, including approach and departure, as well as mating‐related interactions (copulation, female sexual solicitation, and female mating rejection). For consorted females, we also recorded interactions between their consorted partner and rival females, including mating behavior, male partner sexual motivation toward rival females (genital inspection, sexual chasing, and grimacing), and affiliation toward rival females (social grooming and bridging). For relative definitions, see Table 1.

**TABLE 1:** Behavioral definitions

Catalog	Definition
Social interaction	
Approaching	An individual moves within 2 m of another individual
Departure	An individual moves out of the area within 2 m of another individual
Depart distantly	An individual moves out of the area within 5 m of another individual. This refers specifically to female response toward interactions between her consortship partner and rival females
Grooming	One individual manipulates the fur of another individual using his/her hand or mouth, sometimes eating small items found in the fur
Bridging	Two individuals sit face to face, holding an infant between them as the infant lies on its back. The pair usually lick the genitals of the infant while teeth‐chattering vigorously
Reproductive behavior	
Grimacing	A male directs a grimace toward a receptive female that may be either nearby or far away. The grimace differs from a fear grimace in that the corners of the mouth are not drawn back as far. This is a behavior that adult males use to attract receptive females for mating
Copulation	A male mounts a female with intromission and thrusting, but not necessarily ejaculation
Sexual solicitation	A receptive female approaches and presents to an adult male
Mating rejection	A female refuses to copulate with a male, either remaining seated or moving away
Genital inspection	An individual touches the vagina of a female and sniffs it or licks it directly
Sexual chasing	An adult male attempts to mate with a receptive female; if the female refuses and moves away, the male chases her and attempts to mate again

Note

Reproductive behavior and social interaction definitions are modified from Ogawa (1995) and Li and Kappeler (2020).

Behavioral sequences were recorded as follows: (a) approach by either the partner or a rival female; (b) an interaction between the partner and a rival female; (c) the response of the focal female; (d) proximity or interaction termination between the partner and a rival female (either individual's departure—more than 2 m). We set 5‐s intervals between behaviors occurring during the same sequence.

When subjects were involved in conflicts, focal sampling was suspended and behavior sampling methods were used to record the identities of the aggressor, victim, and supporters (if any), after finishing the behavior sampling, focal sampling was continued. We established an aggression–submission matrix (dyadic conflicts only) to calculate the dominance ranks of the adults using David's score (Gammell et al., 2003; Zhang et al., 2014).

All behavior data were collected by voice recorder (ICD‐PX470, SONY China, Beijing, China).

### 2.3 Data analysis

#### 2.3.1 Dominance rank

We constructed a dominance hierarchy for four separate periods, based on the replacement of alpha males and changes in the number of adult group members (Table 2). Male dominance ranks were determined by David's score based on aggressive–submissive matrix (Gammell et al., 2003; Zhang et al., 2014). During the study periods, 130 aggressive–submissive interactions were recorded among the male adults.

**TABLE 2:** Rank and number changes of adult males in YA1 over four periods spanning two mating seasons

Male ID	Period 1 (2 Aug–15 Nov)	Period 2 (16 Nov–31 Jan)	Period 3 (10 Jul–26 Aug)	Period 4 (27 Aug–31 Jan)
HXM*	1	2	2	3
TG*	2	EMI	EMI	EMI
ZB	3	3	3	4
YRB*	4	4	EMI	EMI
BT	5	5	4	5
DS	6	6	5	6
HM	7	7	6	7
TRG*	8	1	1	2
YCLO*	9	8	7	1
YRQ*	10	EMI	EMI	EMI
ZF	–	–	8	8
HL	–	–	9	9

Note

EMI: Individual who emigrated from the YA1 group.

* Males born into the YA1 group.

#### 2.3.2 Female's response to her consorting male

To test whether females were more likely to approach their male partner and less likely to approach other males during the consorted period than that when females were in the nonconsorted period, female approaching behavior (toward partners and other adult males) was documented by focal sampling during the consorted and nonconsorted periods and calculated as hourly frequency.

We used a paired *t*‐test to compare sexual solicitation of each female toward male partners who were consorting with her (calculated as the proportion of total copulations with male partners which were female‐initiated) and adult males who were nonconsorting with her (calculated as the proportion of total copulations with adult males which were female‐initiated). Similarly, a paired *t*‐test was used to compare mate rejection of each female toward male partners were consorted with her (calculated as the proportion of female rejections when the male partners initiated mating) and adult males were consorting with her (calculated as the proportion of female rejections when the adult males initiated mating).

#### 2.3.3 Female's response to rival females

To examine how female responses were affected by interactions between male consorting partner and the rival female, separated generalized linear mixed models (GLMMs) were run for each type of consorted female response. There were three binomial dependent variables (response/no response): depart distantly from partner, approach to partner, and affiliation toward partner. Five predictor variables were included in each model: (a) social rank of the rival female compared to consorted female; (b) age of the rival female compared to consorted females; (c) maternal kinship between the rival female and consorted female; (d) receptive status of the rival female; and (e) interactions between the consorting male partner and the rival female (i.e., affiliations, mating behavior, and sexual motivation). Female ID and male ID were included as random factors. We also ran another GLMM to analyze the effect of female responses on terminations of interactions between their male partners and rival females. In this model, termination of the interaction was the dependent variable, and the predictor variables were the three types of female response (depart distantly from partner, approach to partner, and affiliation toward partner). Female ID and male ID were included as random factors.

The GLMMs were built using a binomial error structure and the logit link function in R 3.6.3 (R Development Core Team, 2020), using the lmer function of the R package lme4 (Bates et al., 2012). In these models, the factors could have potential problems with collinearity. We checked this using variance inflation factors (VIFs, Field, 2005) with the function vif in the R package car (Fox & Weisberg, 2010). The covariation between predictors can be ignored if VIF values are greater than 1 and less than 10 (Bowerman & O'Connell, 1990; Myers, 1990). In our model, the VIFs of predictor variables were all less than 2. The best models were identified by comparing the corrected Akaike information criterion (hereafter AICc), although the model with the lowest value is the best model (Burnham & Anderson, 2010), the models with two lowest value are also considered to fit the data (Burnham & Anderson, 2002), so we further used the model.avg function of the MuMIn package to calculate the relative variable importance for all predictor variables, and then, we can choose the best‐fitting model by retaining the model that was within two AICc units of the lowest value, and which only contained variables with relative variable importance at least 0.7 (Galipaud et al., 2014).

#### 2.3.4 Effects of female preference

To measure female preference, we calculated the approach index *Ia* = *Na*/(*Na* + *Nd*), in which *Na* is the number of female approaches toward a given male and *Nd* is the number of female departures from a given male (Hinde & Atkinson, 1970). We calculated this index only for consortship dyads with 20 or more approaches and departures in total.

Thus, we used partial correlation to analyze the correlation between female preference and consortship duration/copulation frequency, by controlling for male dominance rank. As a contrast, we also used partial correlation to analyze the correlation between male dominance rank and consortship duration/copulation frequency, by controlling for female preference.

## 3 RESULTS

During the whole study period, a total of 47 dyads were collected to form consortships (the same dyads in different mating seasons were counted as separate dyads), and 31 consortship dyads were detailed recorded (20 or more approaches and departures). Five of these detailed recorded dyads were not observed copulating during our focal sampling.

According to the criterion of determining receptive females, we found that, during the mating season 17/18, most females (13/15) went through a receptive status during the study periods. Most receptive females (11/13, two old females were never found to form consortship) were found to form consortship with adult males. The majority of consortships were found to form with the top three high‐ranking males (fourth‐ranking males were found to form consortships only during two blocks). During the mating season 18/19, two‐third (10/15) females went through a receptive status during the study periods. All these receptive females (10/10) were found to form consortship with adult males. The majority of consortships (97.42%) were found to form with the top four high‐ranking males.

### 3.1 Female's response to consorting male

**FIGURE 2:**
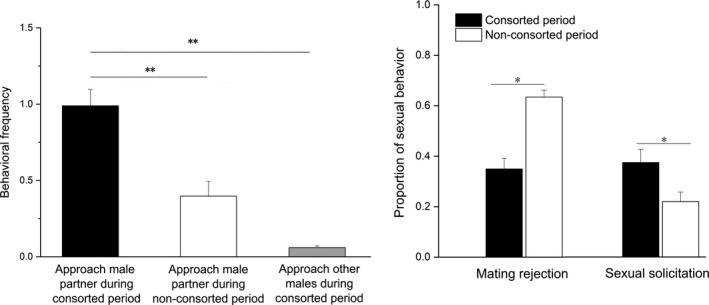
Female cooperative/noncooperative behavior frequency (mean ± *SE*) during 30‐min focal samplings between consorted period and nonconsorted periods. *p < .05, **p < .01

The statistical results showed that females engaged more frequently to approach their consorting male partners during the consorted period than during the nonconsorted period (*t* = 4.046, *n* = 31, *p* = .000). Females were also found to engage fewer approaches to other males than those of their consorting males (*t* = −8.672, *n* = 31, *p* = .000). They also showed less mating rejection (*t* = −5.285, *n* = 12, *p* = .000) and more sexual solicitation (*t* = 2.710, *n* = 12, *p* = .020) toward their consorting partners during the consorted period than when the dyads were not in consorted period (Figure 2).

### 3.2 Female's response to rival females

A total of 537 episodes of approaches were recorded between consorting male partners and rival females, including the episodes that consorting males approached rival females and rival females approached consorting males. Among them, we recorded 115 interactions between males and rival females, including mating, exhibiting sexual motivation, and maintaining affiliative relationships. Consorted females were found to engage in 75 (75/115, 65.22%) episodes of departing distantly from the consorting male‐rival female pair, 10 (10/115, 8.69%) episodes of approach to the consorting male partner, and 20 (20/115, 17.39%) episodes of maintaining affiliative relationships with consorting males.

The best‐fitting model to explain females' depart distantly from their partners included two predictors: female receptive status and interaction between consorting male partner and rival female. However, social rank, age, and maternal kinship of the rival females were not predictors to explain those relationships in this model (Table 3, model 1). We found that consorted females were more likely to depart distantly when the rival female was in a receptive period; Moreover, when male mated or exhibited sexual motivation to rival females, consorted females were found to depart distantly more frequently than that of engaging in nonsexual interactions (such as maintaining affiliative relationships between males and rival females).

**TABLE 3:** Best fitting GLMMs for explaining the responses of consorted females to rival female reproductive state and interactions between their male partner and rival females

Dependent variable (female response)	Independent variable	Estimate	*SE*	*Z*	*p*
Female depart distantly (model 1: AICc = 384.28)	Intercept	0.0788	0.02538	3.100	.002
	Receptive status (rival female): receptive^a^	0.1317	0.0423	3.110	.002
	Affiliation between male and rival female (vs. no interaction)	0.0697	0.0753	0.923	.356
	Male mating with rival female (vs. no interaction)	0.2360	0.0800	2.944	.003
	Male exhibit sexual motivation toward rival female (vs. no interaction)	0.1896	0.0543	3.488	.000
Female affiliation with male partner (model 2: AICc = 145.2)	Intercept	0.0315	0.0297	1.061	.289
	Rank (rival female): lower^b^	0.05615	0.0220	2.542	.011

^a^ Compared with nonreceptive rival females.

^b^ Compared with focal female.

The best‐fitting model to explain the female approach to male partner did not include any predictor variables, so we did not analyze it. The best‐fitting model to explain the affiliation of the consorted females with their male partners included only one predictor: social rank of the rival females. However, age, maternal kinship, receptive status, and interactions with consorting males of the rival females were not predictors to explain those relationships in this model (Table 3, model 2). The consorted females were more likely to form affiliative relationships with their consorting males to reduce the opportunities that the rival female approached and established affiliative relationships with these males when the social ranks of consorted females were higher than those of the rival females.

Further analysis using GLMM showed that female depart distantly was a significant predictor to terminate the interactions between the consorting male partner and the rival female. The interactions of consorting males with the rival females decreased when the consorted females departed distantly from them (Table 4).

**TABLE 4:** GLMM binomial regression results for the relationship between female responses (compared with no responses) and the terminations of interactions between their male partners and rival females (separated by at least 5 m)

Independent variable	Estimate	*SE*	*Z*	*p*
Intercept	−2.1518	0.1575	−13.662	<.001
Affiliation	0.4172	0.6457	0.646	.518
Approach	1.3045	0.7078	1.843	.065
Distance departure	2.1784	0.2796	7.793	<.001

### 3.3 Effects of female preference

**FIGURE 3:**
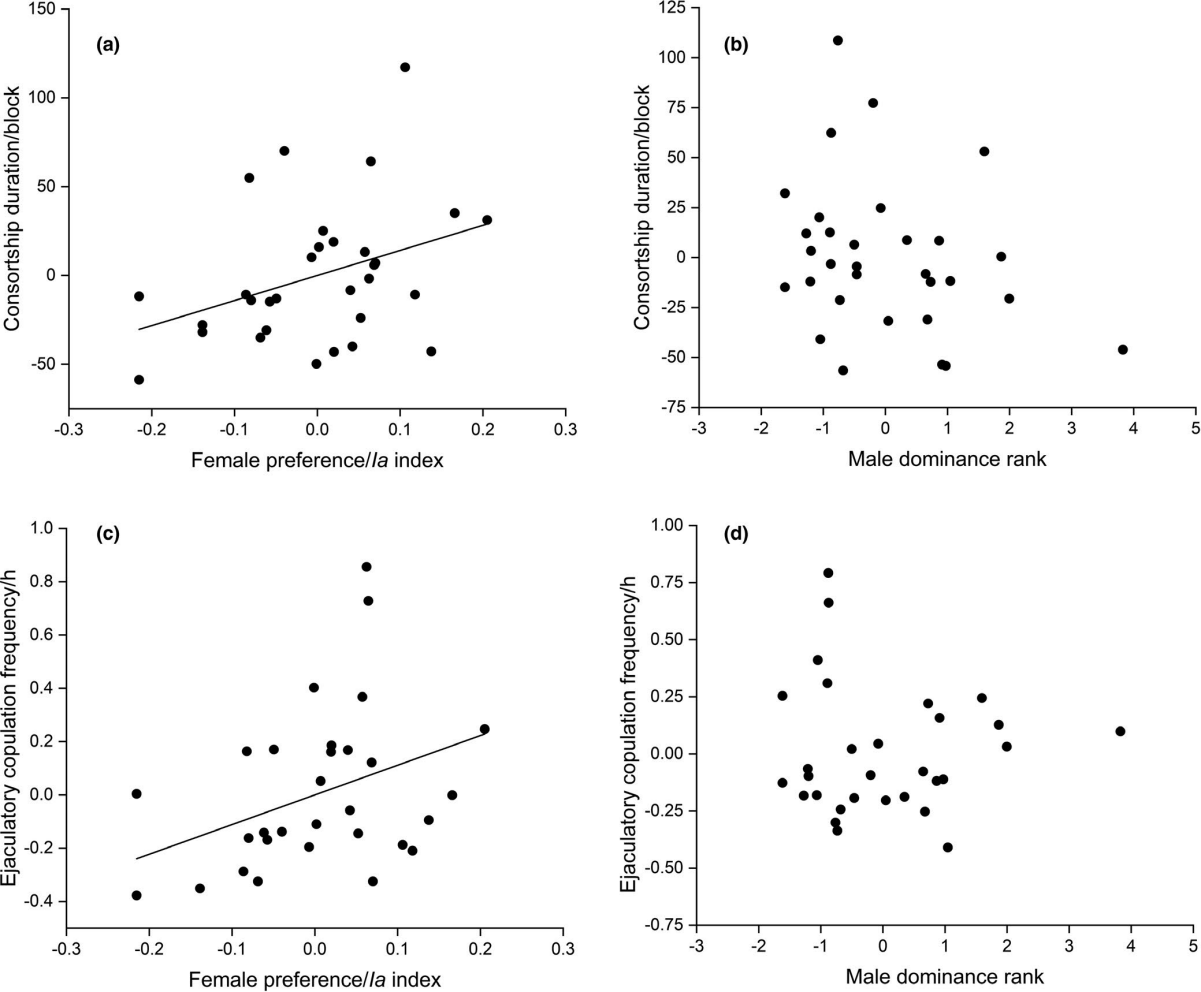
Effects of (a) female preference on consortship duration by controlling male dominance rank, (b) male dominance rank on consortship duration by controlling female preference, (c) female preference on ejaculatory copulation frequency by controlling male dominance rank, and (d) male dominance rank on ejaculatory copulation frequency by controlling female preference. The consort duration was measured by adding every block; the dyads were recorded as consortship. The ejaculation frequency was calculated by the hourly frequency of each dyad during the 30‐min focal samplings. Values of consortship duration and ejaculatory copulation frequency are based on residual values derived from regression models

Partial correlation tests controlling for male dominance rank showed that female preference is strongly linked to the duration of consortship (Rs = 0.364, *p* = .048, Figure 3a) and the frequency of ejaculatory copulation (Rs = 0.376, *p* = .041, Figure 3c). However, after controlling for female preference, the male dominance rank had relatively little impact on the duration of consortship (Rs = −0.274, *p* = .135, Figure 3b) or the frequency of ejaculatory copulation (Rs = −0.029, *p* = .877, Figure 3d).

## 4 DISCUSSION

Consortship has been widely used to explain the behavioral strategies of adult males to facilitate male mating efforts, male mate guarding, mating success, and reproductive success. Using the responses of consorted females to consorting males, the rival females, and the preference of consorted females, we investigated the female behavioral strategies during the consortships. Our results showed that female Tibetan macaques were more likely to approach the consorting males during the consorted period than during the nonconsorted period, and exhibit sexual solicitation to their consorting males. In addition, consorted females were more likely to depart distantly when their consorting male partners mated with or showed sexual motivation toward other rival females. Moreover, the duration of consortship and the frequency of ejaculation copulation during the consorted period related the female preference. It indicated that, apart from male strategies, female strategies also played critical roles in forming and maintaining consortship. It provided new insight into understanding female adaptive strategies to male strategies during consortships in multimale–multifemale primate species that females concealed their ovulation.

Our results showed that females approached and showed sexual solicitation to their consorting males during the consorted period, and females also exhibited less approach to their nonconsorting males. The severity of male–male competition and female mate choice may help to explain why female strategies evolved during the period of being consorted by adult males. In the primate species with despotic style living in multimale and multifemale society, males preferred to mate with high rank, high fecundity, and middle‐age females (Zhang et al., 2010). However, only high‐ranking males have the chance to mate with multiple estrous females and increase reproductive success (Altmann, 1962; Dubuc et al., 2011; Li et al., 2005). Previous studies also demonstrated that females preferred to mate with high‐ranking males (Small, 1989) to get food resources, social support, paternal care, and so on (Andersson & Simmons, 2006; Paul, 2002). As such, female mate choice might be considered as a potentially powerful selective force to drive the adaption of intersexual social relationships. For example, in the species that females show obvious sexual signal (i.e., with sexual swelling during the estrous period), such as Tonkean macaques (*Macaca tonkeana*) and Barbary macaques (*Macaca sylvanus*), females provided the clear sexual signal to improve the opportunities of mating with high‐ranking males (Brauch et al., 2008; Fitzpatrick et al., 2015; Thierry et al., 1996). However, in the species that females conceal their ovulation (i.e., without sexual swelling), such as seasonal breeding Tibetan macaques, females were receptive synchronously during the mating season, and adult males could not identify the female's fertile phase (Li et al., 2005). In these primate species, female mate choice might play crucial roles in forming intersexual relationships (Manson, 1992, 2007). Tibetan macaques form a rigid linear dominance hierarchy, and more than two‐thirds copulations of the whole group were found to be monopolized by the three highest social rank males (Li, 1999). In this study, the majority of consortships (>95%) were found to form by the adult males with the top four highest social rank. To reduce the potential opportunities of behavioral interactions between high‐ranking males and rival females, females might show positive responses to their consorting males (i.e., approached frequently and exhibited more sexual solicitation). In contrast, in rhesus macaques (*Macaca mulatta*), females were compelled to respond to a high‐ranking male's guarding by maintaining proximity to this male (Manson, 1992).

In addition, we also found that females would not exhibit strong responses when their consorting males maintained affiliative relationships with rival females, whereas females strongly responded (either departed distantly or formed affiliative relationships with their consorting male partner) when their consorting males mated with rival females or showed sexual motivation to rival females. It indicated that the sexual information between consorting males and rival females could be the critical factor to influence the consortship. The processes of forming and maintaining consortship may help to explain why females strongly responded when their consorting males showed sexual interactions with rival females. In Tibetan macaques, the consorted female partner of an adult male often changed from female A to female B after occasional copulation between this adult male and female B (personal observation, QXZ). And thus, females would evolve behavioral strategies to prevent them from mating with rival females or attempt to terminate the sexual relationships between their consorting males and rival females. Our results suggested that females would maintain affiliative relationships with their consorting males to decrease the mating opportunities with rival females of their consorting males when the social ranks of rival females were lower. Moreover, females would change their behavioral strategies when their consorting males showed sexual motivation or mated with the rival females. In some species, such as Assamese macaques (*Macaca assamensis*), a relatively tolerant social style and lower male monopolization, Sukmak et al. (2014) suggested that adult males would more likely to move on to another female when their consorted females were uncooperative during the consorted period. However, in most species with despotic social style, a high‐ranking male would follow “ensure” strategy by extending their consort behavior by consistently following one receptive female (*Macaca fuscata*, Inoue et al., 1991; *Macaca mulatta*, Manson, 1997; *Macaca fasciculari*s, Girard‐Buttoz et al., 2014). In this study, during all the consortships within 31 intersexual dyads, all the adult male Tibetan macaques were found to continuously follow their consorted females frequently when females departed distantly away (Zhang et al., unpublished data). Accordingly, a female's departure distantly from her consorting male partner would be an alternative strategy to get his attention, resulting in the prevention of potential mating behavior or the termination of social relationships after the copulation between the consorting male and rival female.

Moreover, our results showed that female preferences were positively correlated to the duration of consortships and the frequencies of ejaculation copulations, independent of the social ranks of their consorting male partners. It suggests that female efforts played crucial roles in maintaining consortship and benefiting by maintaining these intersexual social bonds. Similar results have been reported in several primate species that females do not show obvious estrous statuses, such as Japanese macaques, rhesus macaques, and Assamese macaques (Huffman, 1992; Manson, 1992; Sukmak et al., 2014). There was evidence that males would likely form consortship and successfully prevented fertile females from expressing direct mate choice when females showed obvious sexual swelling and males could identify female fertile phases, such as mandrills, baboons, and Tonkean macaques (Clarke et al., 2009; Noë & Sluijter, 1990; Rebout et al., 2017; Setchell et al., 2005; Weingrill et al., 2003). However, in species that females conceal their ovulation, males were limited to recognize female's reproductive status, and thus, some authors proposed that the concealed ovulation might allow females to better exercise their mating choice (Manson, 2007). Our results provided supportive evidence.

Female mate choice can bring several direct benefits (i.e., resources), which have been demonstrated in many nonhuman primates. One typical example is that females can trade sex for food, which has been reported in bonobos (*Pan paniscus*), chimpanzees (*Pan troglodytes*), and rhesus macaques (*Macaca mulutta*) (Dubuc, Hughes, et al., 2012; Goodall, 1986; Kuroda, 1984). Besides, females might gain social protection by mating with high‐ranking males; for example, in Tibetan macaques (*Macaca thibetana*), by copulating with a high‐ranking male, females can obtain the male's agonistic support against their opponent (Wang et al., 2013). In addition, females might prefer to mate with multiple males to confuse paternity or increase paternal care; for example, in Barbary macaques (*Macaca sylvanus*), females tend to mate with multiple males to gain paternal investment from more than one male (Bissonnette et al., 2011). Nonetheless, Paul (2002) proposed that “often, females get nothing more from a copulation than the male's sperm,” and thus, researchers assumed that female mate choice might benefit their offspring fitness in terms of genetic by mating with an attractive male. Accordingly, several theories have been assumed to associate male attractiveness and offspring fitness, such as Fisher's “runaway process” and “good genes” models (Summarized from Paul, 2002), though these theories are still controversial.

In conclusion, our study provides new insight into understanding the importance of female strategies during the consorted period in promiscuous mating systems. It suggests that in the species with that females conceal their ovulation, males might not identify the female's fertile phase and extended the duration of consortship. It provides an ideal opportunity for females to exhibit their behavioral strategies and positively respond to the male mate guarding. It would facilitate females to obtain potential benefits when they were consorted by high‐ranking males. Future studies should pay more attention to the relationships between consortship and male mating success to increase the potential reproductive success, and the benefits obtained of consorted females, such as intrasexual mating competition success, the survivorship of their offspring, and social tolerance. These studies would expand our knowledge to understand the evolution of consortship in multimale and multifemale society.

## CONFLICT OF INTEREST

None declared.

## AUTHOR CONTRIBUTIONS

**Qi‐Xin Zhang:** Data curation (lead); formal analysis (equal); investigation (lead); methodology (equal); supervision (equal); writing‐original draft (lead); writing‐review & editing (equal). **Lixing Sun:** Methodology (supporting); writing‐original draft (supporting); writing‐review & editing (supporting). **Dong‐Po Xia:** Conceptualization (lead); formal analysis (equal); writing‐review & editing (equal). **Jin‐Hua Li:** Conceptualization (lead); funding acquisition (lead); resources (lead); supervision (equal); visualization (equal); writing‐review & editing (equal).

### OPEN RESEARCH BADGES

This article has earned an Open Data Badge for making publicly available the digitally‐shareable data necessary to reproduce the reported results. The data is available at https://doi.org/10.6084/m9.figshare.13311746.

## Data Availability

The dataset generated and analyzed during the current study is available in the open figshare repository, https://doi.org/10.6084/m9.figshare.13311746
